# Recent Research Progresses and Challenges for Practical Application of Large-Scale Solar Hydrogen Production

**DOI:** 10.3390/molecules29246003

**Published:** 2024-12-20

**Authors:** Min-Kyu Son

**Affiliations:** Nano Convergence Materials Center, Emerging Materials R&D Division, Korea Institute of Ceramic Engineering & Technology (KICET), Jinju 52851, Republic of Korea; minkyu.son@kicet.re.kr; Tel.: +82-55-792-2683

**Keywords:** solar hydrogen production, large-scale system, commercialization, photovoltaic electrolysis, photoelectrochemical, photocatalyst

## Abstract

Solar hydrogen production is a promising pathway for sustainable CO_2_-free hydrogen production. It is mainly classified into three systems: photovoltaic electrolysis (PV-EC), photoelectrochemical (PEC) system, and particulate photocatalytic (PC) system. However, it still has trouble in commercialization due to the limitation of performance and economic feasibility in the large-scale system. In this review, the challenges of each large-scale system are, respectively, summarized. Based on this summary, recent approaches to solving these challenges are introduced, focusing on core components, fabrication processes, and systematic designs. In addition, several demonstrations of large-scale systems under outdoor conditions and performances of upscaled systems are introduced to understand the current technical level of solar-driven hydrogen production systems for commercialization. Finally, the future outlooks and perspectives on the practical application of large-scale solar-driven hydrogen production are discussed.

## 1. Introduction

Hydrogen plays a critical role in the realization of a carbon-neutral society for human survival because it is a clean energy carrier. However, paradoxically, 99% of global hydrogen production depends on fossil fuels at present [1]. It emits huge amounts of CO_2_, one of the greenhouse gases, thereby having a negative impact on a carbon-neutral society. Therefore, it is essential to explore sustainable CO_2_-free hydrogen production technology to meet net-zero CO_2_ emissions by 2050. Solar-driven hydrogen production has been in the limelight as a promising route for sustainable CO_2_-free hydrogen production because it generates hydrogen and oxygen via water-splitting reactions triggered by sunlight without CO_2_ emission [2,3,4].

**Figure 1:**
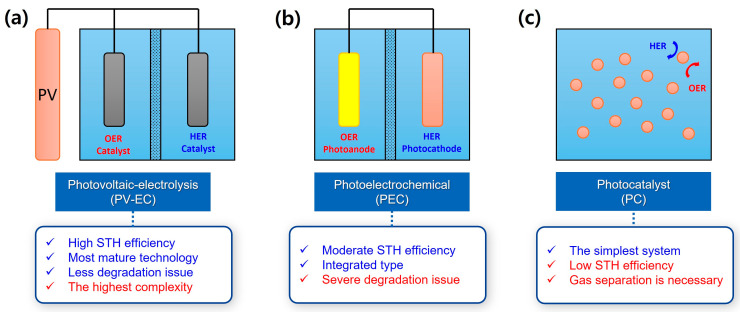
Solar-driven hydrogen production systems: (**a**) photovoltaic-electrolysis (PV-EC), (**b**) photoelectrochemical (PEC), and (**c**) particulate photocatalytic (PC) system.

There are three main systems in solar-driven hydrogen production: photovoltaic-electrolysis (PV-EC) [5,6], photoelectrochemical (PEC) system [7,8,9,10], and particulate photocatalytic (PC) system [11,12,13], as illustrated in Figure 1. They have their own pros and cons. First, in the PV-EC system, only catalysts for hydrogen evolution reaction (HER) and oxygen evolution reaction (OER) are soaked in the water, while photovoltaic (PV) cells are located outside the water. Hence, the degradation issue is minimized. In addition, it is a mature technology because its efficiency is relatively high due to the advancement of PV technology. However, it is expensive due to its complexity. Second, the PEC system is an integrated system because the light utilization and water splitting reaction occur at the same photoelectrode in the water. On the other hand, its stability is poor because most PEC materials are prone to corrode in the aqueous solution. Third, the particulate PC system is the simplest system because hydrogen and oxygen are simply generated from the dispersion of PC powders in the water. Although it is simple, its efficiency is relatively low. Furthermore, in terms of safety issues, the specific strategy to separate generated hydrogen and oxygen from PC powders is necessary because there is no membrane in this system.

Each solar-driven hydrogen production system has achieved a splendid development based on their respective advantages since the discovery of the electrochemical photolysis effect by Fujishima and Honda in 1972 [14]. As a result, its solar-to-hydrogen (STH) efficiency has dramatically improved up to 30% [15], which surpasses the minimum STH efficiency (20%) for the commercially viable system recommended by the US Department of Energy (DOE) [16]. However, it is limited to the lab scale device with an active area below 1 cm^2^. It is still low in the large-scale devices for mass production of hydrogen. In addition, its stability is not sufficient for the long-term operation. Hence, it is still challenging to utilize it in the practical application for sustainable hydrogen production. It is reported that the STH efficiency should be higher than 6%, while it should be maintained within a 10% decrease during the continuous operation for at least 6 months to achieve the levelized cost of hydrogen production via solar hydrogen production [17]. Therefore, it is inevitable to further improve the performance and stability of large-scale systems to be competitive with other hydrogen production techniques.

In this review paper, recent efforts on the development of large-scale solar-driven hydrogen production systems focusing on three main systems (PV-EC, PEC, and particulate PC systems) are thoroughly examined. In detail, major obstacles to the efficient and durable large-scale system are, respectively, summarized. Furthermore, various strategies to overcome them are introduced in terms of core components, fabrication processes, and design issues. The demonstrations and outdoor test results of large-scale solar-driven hydrogen production systems are also summarized to understand the current technical level for commercialization. Finally, future outlooks and perspectives on the real-life application of large-scale solar-driven hydrogen production for sustainable CO_2_-free hydrogen production toward a carbon-neutral society are discussed. It will provide us with comprehensive and realistic strategies for the commercialization of the solar hydrogen production system in the future.

## 2. Challenges and Research Progresses

### 2.1. PV-EC System

In the PV-EC system, PV cells provide the driving force to split water by generating electron-hole pairs when the light is illuminated. The illuminated PV cell should have a high voltage above 1.7 V, at least considering the thermodynamic water splitting potential (1.23 V) and overpotentials for HER/OER reactions [18,19]. Silicon (Si) PVs are widely used in the present PV market because they show high efficiency and considerable durability in large-scale applications. To complete the overall water-splitting reaction, single or double-junction Si PV cells should be connected in series due to the insufficient voltage (0.6~0.7 V) [20]. Multijunction Si PV cells are a promising option to provide sufficient voltage for overall water splitting without the series connection. The triple junction Si PV cell with hydrogenated amorphous silicon (a-Si:H)/a-Si:H/hydrogenated microcrystalline silicon (μc-Si:H) shows a sufficient voltage above 2.0 V. Thus, a-Si:H/a-Si:H/μc-Si:H triple junction Si PV modules are extensively applied to the large-scale PV-EC system in the present [21,22,23].

Other PV cells are also utilized for the large-scale PV-EC system. Cu(In,Ga)Se_2_ (CIGS) PVs were applied to the large-scale PV-EC system as a monolithically interconnected module [24,25]. They showed considerable STH efficiency above 10% even in the large area system around 100 cm^2^. However, high temperature is necessary to fabricate the CIGS thin film with good quality, which is unfavorable to the large-scale module in terms of energy consumption in the fabrication process. III-V semiconductor PVs were successfully demonstrated for achieving the high performance of PV-EC systems [26,27,28]. Nevertheless, it has a cost issue in the large-scale device because some materials such as Ga, As, and Te are expensive, and a subsidiary facility such as a solar concentration system is necessary. On the other hand, perovskite solar cells are a prominent candidate for the low-cost, large-scale PV-EC system. Yu et al. used a perovskite module as a source of driving force for water-splitting reactions [29]. It produced a small amount of hydrogen (1.5 mL) during the operation for 5 min, with graphite HER/OER catalysts. However, the size of a perovskite module (12.6 cm^2^) was still relatively small compared to other PV modules. Therefore, it is essential to further increase the module size along with the high efficiency by mitigating the current loss and fabricating the uniform perovskite layer in the large area for the practical application of the PV-EC system.

**Figure 2:**
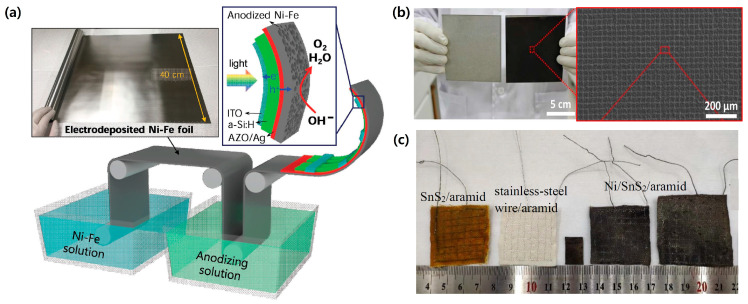
Scalable catalyst electrode for large-scale PV-EC system. (**a**) Schematic illustration of roll-to-roll fabrication process of anodized Ni-Fe OER catalytic substrate. Reprinted from [30] with permission from Wiley-VCH Verlag GmbH & Co. (Weinheim, Germany), copyright 2017. (**b**) NiO hierarchical nanostructured bifunctional catalyst electrodes fabricated by pulsed laser ablation. Reprinted from [31] with permission from Elsevier Ltd. (Amsterdam, The Netherlands), copyright 2017. (**c**) Ni bifunctional catalyst on the fabric electrode with the assistance of solvent-thermally deposited SnS_2_ thin film and stainless steel (SS) mesh. Reprinted from [32] with permission from the Royal Society of Chemistry, copyright 2024.

Catalysts are also a key component in the PV-EC system. They play a critical role in the improved performance of the PV-EC system by reducing HER/OER overpotentials. In the large-scale PV-EC system, it is important to fabricate durable large-area catalyst electrodes with highly catalytic active sites. In general, catalyst powders are deposited on the conductive substrate with the assistance of a binder. However, it does not guarantee high catalytic performance and long-term durability because the charge transport is low due to the binder and catalysts being detached from the substrate during the operation. Many efforts have been carried out to solve these challenges on the large-area catalyst electrodes. Dong et al. fabricated a scalable Ni-Fe-based OER catalyst electrode by the anodization of electrodeposited Ni-Fe foil [30]. It showed excellent catalytic performance and stability in the strong alkaline solution. In addition, it is easily scalable by the roll-to-roll process, as illustrated in Figure 2a. Ou et al. reported NiO hierarchical nanostructured bifunctional catalyst electrodes [31]. Nanostructured NiO with many oxygen defects was formed on the Ni plate by pulsed laser ablation (Figure 2b). Furthermore, its surface was hydrophilic, thereby improving the contact with the electrolyte. It accelerated the catalytic performance on the large-area electrode. Yuan et al. suggested a flexible fabric as an alternative to the supporting substrate for the large-area electrode [32]. They successfully deposited Ni bifunctional catalysts on the aramid fabric electrode with the assistance of solvent-thermally deposited SnS_2_ thin film and stainless steel (SS) mesh (Figure 2c). The SnS_2_ thin film enhanced the adhesive force of catalysts on the fabric electrode, while the SS mesh improved the conductivity of the fabric electrode. It is beneficial for reducing the fabrication cost of catalyst electrodes in the large-scale PV-EC system.

**Figure 3:**
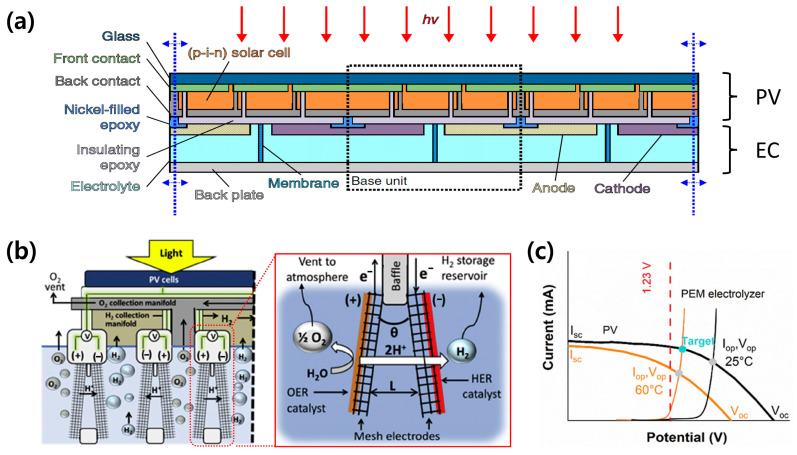
Various configurations and characteristics of PV-EC system. (**a**) Integrated PV-EC system. Reprinted from [19] with permission from The Authors, copyright 2016. (**b**) Floating membrane-less PV-EC system with tilted asymmetric electrodes. Reprinted from [33] with permission from Elsevier Ltd., copyright 2018. (**c**) Performance of PV and electrolyzer in the thermally integrated PV-EC system depending on the temperature. Reprinted from [35] with permission from the Royal Society of Chemistry, copyright 2024.

System configuration and design should be considered for the practical application of the PV-EC system. In the traditional PV-EC system, PV cells and catalyst electrodes are externally connected using wires. To simplify this, an integrated PV-EC system was introduced, as illustrated in Figure 3a [19]. It is a compact design that is economically viable because it does not need wires and power converters. However, a membrane, which is one of the expensive components, is still necessary to separate the generated hydrogen and oxygen in this system. The floating membrane-less PV-EC design suggested by Davis et al. is a feasible option to further reduce the cost of the PV-EC system [33]. As illustrated in Figure 3b, it had tilted asymmetric electrodes with the selective placement of HER/OER catalysts. This unique design induced the gas separation by buoyance, enabling the PV-EC system to separate the generated hydrogen and oxygen without membrane. It was expected that it would be applicable to the floating PV modules or the seawater electrolysis in the future. The combination of PV modules with traditional electrolyzers such as proton exchange membrane (PEM) [34,35] and anion exchange membrane (AEM) [36] systems provides more realistic solutions for the practical PV-EC system. The PV-PEM system is suitable for the management of the fluctuation of solar energy, while the PV-AEM system is reasonable for the highly efficient and low-cost system. It is possible to improve its efficiency by heat management when two components are integrated using boron nitride thermal paste or indium sheet. However, the trade-off of temperatures in the PV and electrolyzer is essential for the efficient system because the heat causes the degraded performance of PV, while it results in the improved performance of the electrolyzer (Figure 3c).

Table 1 summarizes the performance of large-scale PV-EC systems. The PV-EC system with III-V semiconductor PV and Pt HER/IrO_2_ OER catalysts shows the highest STH efficiency (20.3%), which meets the standard for commercialization at the US DOE. However, it is unfavorable to complete the economic PV-EC system because its components, such as III-V semiconductor, Pt, and IrO_2_, are expensive. In addition, it needs additional facilities such as a collecting mirror because it uses concentrated sunlight. Although earth-abundant materials have been applied to HER/OER catalysts, expensive materials, such as Pt and Ir, are still dominant in the catalyst parts of the PV-EC system. Hence, the development of low-cost HER/OER catalysts with highly active catalytic performance is inevitable for reducing the manufacturing cost of large-scale PV-EC systems.

### 2.2. PEC System

Photoelectrodes play a significant role in the PEC water-splitting system. Electron-hole pairs are generated in the photoelectrode when the light is illuminated. Then, they participate in the water-splitting reaction at the interface between photoelectrode and water. In general, oxygen is generated from the photoanode based on n-type semiconductors, while hydrogen is generated from the photocathode based on p-type semiconductors [38]. The main challenge for the large-scale PEC system is a huge decrease in PEC performance in the large area photoelectrode. It is mainly caused by the resistive loss of the large area substrate, the non-uniformity of upscaled photoactive film, and the limited ionic conductivity [39,40,41]. Therefore, a resolution of these limitations is a prerequisite for the commercialization of large-scale PEC systems.

**Figure 4:**
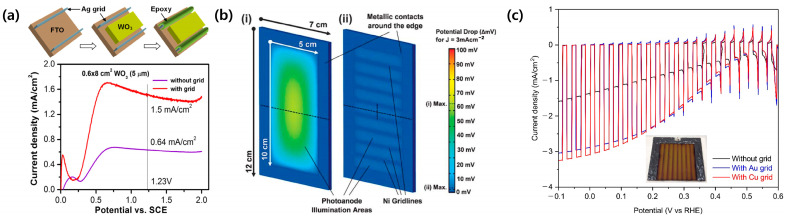
Metal grid structure inserted large-scale PEC photoelectrodes. (**a**) Effect of Ag grid lines on the PEC performance of large scale WO_3_ photoanodes. Reprinted from [44] with permission from Elsevier Ltd., copyright 2011. (**b**) Comparison of a voltage drop in the upscaled BiVO_4_ photoanode with and without Ni busbar lines. Reprinted from [39] with permission from the Royal Society of Chemistry. (**c**) Improved PEC performance of a 25 cm^2^ Cu_2_O photocathode with Au and Cu grid lines. Reprinted from [45] with permission from The Author, copyright 2021.

Transparent conductive substrates, such as indium-doped tin oxide (ITO) and fluorine-doped tin oxide (FTO) glasses, have been generally used as a substrate for PEC photoelectrodes because they facilitate the sufficient penetration of light to the photoactive film. However, it is not suitable for the efficient large-scale PEC photoelectrode due to its high sheet resistances (7~16 Ω/sq). It causes a severe voltage drop, resulting in poor PEC performance in the upscaled photoelectrode [42,43]. The metal grid structure is valuable for reducing the ohmic resistance in the large area substrate. In addition, it also induces the uniform deposition of photoactive film on the large area substrate. Lee et al. introduced screen-printed Ag grids on the large-scale WO_3_ photoanode with an active area of 130.56 cm^2^ [44]. The PEC performance was more than double, compared to one without Ag grid lines (Figure 4a). Ahmet et al. successfully fabricated a 50 cm^2^ BiVO_4_ photoanode with Ni busbar lines [39]. It was demonstrated that the Ni busbar lines are effective in reducing the voltage drop in the large area photoelectrode, resulting in improved PEC performance (Figure 4b). Au and Cu grid lines were also applied to a 25 cm^2^ Cu_2_O photocathode [45]. As shown in Figure 4c, the device showed an improved PEC performance, especially photocurrent density and fill factor, owing to the better conductivity. However, the appropriate protection on the metal grid structure is necessary for the durable PEC system because most metals are easily corroded in the water, thereby losing their intrinsic conductivity.

The usage of substrates with high conductivity is an alternative route for reducing the resistive loss in the upscaled photoelectrode. SS substrate is a promising candidate because it is more conductive than transparent conductive glass substrates. In addition, it is robust to the corrosion in the aqueous solution. Shinde et al. successfully fabricated a large-scale WO_3_ photoanode on the SS sheet with an active area of 81 cm^2^ by the screen print method [46]. Moreover, they further improved the photocurrent density of the WO_3_ photoanode by introducing the SS mesh substrate. Deposited WO_3_ film by brush painting on the SS mesh substrate provided a high surface area, resulting in enhanced PEC performance. Recently, the Ti porous transport layer was also used as a substrate by Kunturu et al. [47]. They fabricated a 100 cm^2^ BiVO_4_ photoanode on the Ti felt by a successive ionic layer adsorption and reaction method. Its PEC performance maintained almost 90% of the PEC performance in small-sized BiVO_4_ photoanodes (1 cm^2^), even if the area is 100 times larger. It was due to the quite low sheet resistance of Ti felt (0.048 Ω/sq), compared to the FTO substrate.

**Figure 5:**
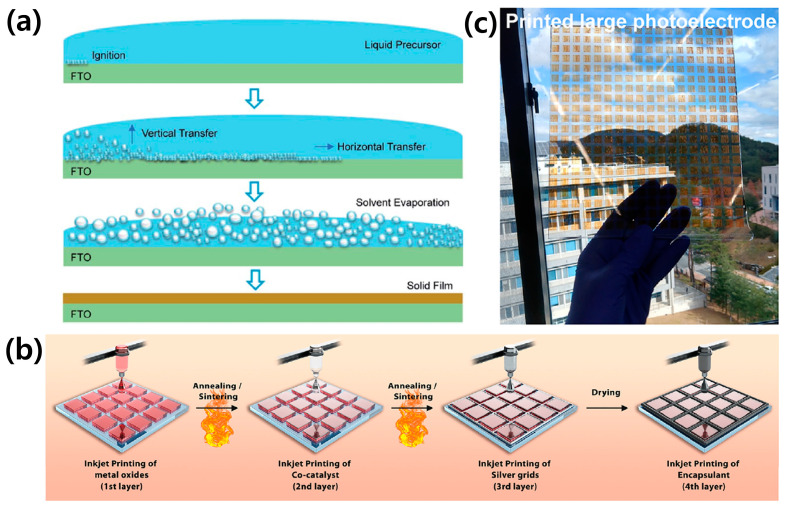
Novel deposition methods for the uniform large-scale photoelectrode. (**a**) Schematic illustration of the combustion synthesis process of large-scale BiVO_4_ photoanode. Reprinted from [53] with permission from the Royal Society of Chemistry, copyright 2020. (**b**) Schematic illustration of inkjet printing method. (**c**) Large-scale Fe_2_O_3_ photoanode array with an illumination area of 500 cm^2^ fabricated by the inkjet printing method. Reprinted from [54] with permission Elsevier Inc., copyright 2023.

It is possible to ameliorate the non-uniformity of upscaled photoactive films by adjusting the film deposition process. Spray pyrolysis deposition has been widely used for depositing uniform large-scale photoelectrode without defects because it yields homogenous film with high crystallinity [39,48,49,50]. Furthermore, it is easily scalable because it is simple and inexpensive. Metal-organic decomposition (MOD) deposition is also suitable for homogeneous large-scale photoelectrode, especially BiVO_4_ photoanode [51,52]. Qayum et al. developed a novel deposition method to improve the adhesion of photoactive film to the substrate [53]. They deposited the precursor solution on the substrate by spray coating. The film was completed by evaporating the precursor solution via a sequential combustion process. The fast precursor evaporation induced by the short heat treatment prevented the aggregation of the photoactive film (Figure 5a). As a result, they successfully upscaled the BiVO_4_ photoanode with an excellent uniformity up to 100 cm^2^. On the other hand, the inkjet printing method suggested by Hansora et al. is useful for the mass production of large-scale photoelectrode due to its fast and simple processing steps [54]. It was possible to efficiently fabricate the thin-film large photoelectrode device with co-catalysts, a metal grid, and encapsulation (Figure 5b). Consequently, the large-scale Fe_2_O_3_ photoanode arrays with an illumination area of 500 cm^2^ were demonstrated, as shown in Figure 5c.

Modifications of the deposition process are useful to control the uniformity of large-area photoactive film. Kazemi et al. controlled the uniformity of BiVO_4_ photoanode by adjusting the viscosity and wettability of the precursor [55]. They improved the viscosity of the precursor for the spin coating by introducing industrial ethylene glycol (EG). They also enhanced the wettability with a preliminary heat process before the spin coating. The preheat temperature around 200 °C accelerated the fast evaporation of EG in the precursor, thereby forming a homogeneous and pinhole-free BiVO_4_ film. They successfully scaled up the uniform BiVO_4_ photoanode without any pinholes up to 58 cm^2^. Meanwhile, it is difficult to fabricate homogeneous large-scale perovskite photoelectrodes due to the inhomogeneous nucleation of perovskite during the film deposition. To overcome this challenge, Jeong et al. suggested a dipping method in an antisolvent with a passivation additive [56]. As a result, the uniform perovskite film without defects at grain boundaries was fabricated due to the fast nucleation of perovskite triggered by the passivation additive. They successfully demonstrated a large-scale unbiased PEC water-splitting device with an STH efficiency of 3.09% using this perovskite photoelectrode with an area of 100 cm^2^.

**Figure 6:**
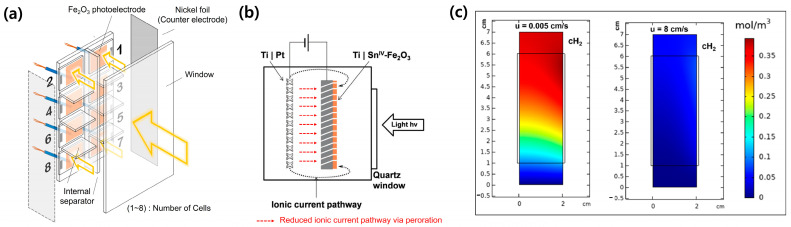
Designs and effects of large-scale PEC devices and reactors. (**a**) Schematic illustration of multi-photoelectrodes installed in a segmented support reactor. Reprinted from [57] with permission from Elsevier Ltd., copyright 2018. (**b**) Effect of perforated photoelectrodes on the reduction of ionic current pathway. Reprinted from [58] with permission from the Royal Society of Chemistry, copyright 2017. (**c**) The concentration of hydrogen under different flow rates of electrolyte. Reprinted from [41] Copyright 2023 American Chemical Society.

On the other hand, the design of PEC devices or reactors has been considered to alleviate the limited ionic conductivity. Vilanova et al. proposed a multi-photoelectrode concept for the large-scale PEC system [57]. In their concept, eight small pieces of Fe_2_O_3_ photoanodes with an area of 3.2 cm^2^ were installed in a segmented support reactor with internal separators, as shown in Figure 6a. It was demonstrated that it provides efficient ionic paths without the parasitic effect, resulting in the reduction of additional overpotential losses. Its PEC performance was similar to one of small Fe_2_O_3_ photoanodes (3.2 cm^2^), even though the area was increased up to 28 cm^2^. The perforated photoelectrode suggested by Hankin et al. was efficient in reducing the ionic current pathway, as illustrated in Figure 6b [58]. It minimized the imbalance of ionic current distribution in the large-scale photoelectrode. Its effect was more remarkable in the low-concentration electrolyte. Electrolyte flow is indispensable for improving the limited ionic conductivity in the large area photoelectrode [41]. It was proved that it efficiently solves a pH gradient issue limiting by continuously replenishing the H^+^/OH^−^ ions (Figure 6c). In addition, it prevented the accumulation of generated hydrogen/oxygen bubbles on the surface of photoelectrodes, thereby resolving the decreased PEC performance by the light scattering effect.

**Figure 7:**
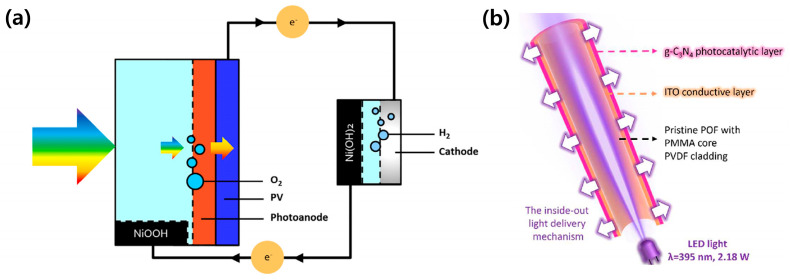
Novel design of PEC system for commercialization. (**a**) Schematic illustration of membrane-free decoupled water-splitting system using nickel hydroxide-based auxiliary electrodes. Reprinted from [59] with permission Elsevier Inc., copyright 2020. (**b**) Schematic illustration of polymer optical fiber incorporated PEC. Reprinted from [60] Copyright 2024 American Chemical Society.

Furthermore, to prepare for the commercialization of the PEC system, a novel design of the system has recently been introduced. Landman et al. proposed a membrane-free decoupled water-splitting system (Figure 7a) using nickel hydroxide-based auxiliary electrodes [59]. In this system, a hydrogen production part was physically separated from the PEC photoelectrode, while oxygen was directly released into the air from the photoanode. Ions were exchanged via reusable auxiliary electrodes, not the membrane. It is beneficial for constructing the large-scale PEC system to be economically viable because its maintenance is easy, and expensive components, such as membrane and gas pipes for transporting hydrogen, are unnecessary. Polymer optical fiber incorporated PEC system suggested by Fu et al. is a compact design for the large-scale PEC system [60]. They fabricated graphite carbon nitride photocathode on poly(methyl methacrylate) core polyvinylidene fluoride cladding (Figure 7b). It was expected that it would be easily scalable with the low space requirement by using a bundle of optical fiber. It is possible to set up a large-scale PEC system in a limited space because it does not need a huge area, contrary to the traditional PEC system based on thin film electrodes.

Table 2 shows the performance of large-scale photoelectrodes for the PEC system. As shown in Table 2, photoanodes have been intensively developed as a large area PEC photoelectrode, while little research on the photocathode has been carried out. Therefore, further efforts on the large-area photocathode are necessary for the large-scale PEC system. On the other hand, the photocurrent density is considerably decreased in the large area photoelectrode above 25 cm^2^, compared to the lab-scale photoelectrode, even though co-catalysts are deposited on the PEC electrodes. Hence, it should be further enhanced by reducing the resistive loss, fabricating homogeneous photoelectrode, and improving the limited ionic conductivity. Completing the overall water splitting through the PEC system requires an unbiased PEC system, such as PEC-PV and PEC-PEC system. Table 3 summarizes the performance of large-scale PEC-PV and PEC-PEC systems operated without any external bias. It is classified into three categories: side-by-side, tandem, and assembled type. The side-by-side configuration is advantageous for utilizing the light because the light is illuminated to all components without any optical losses. On the other hand, the tandem and assembled configurations are beneficial for reducing the ionic loss, but the band gap of the top and bottom absorbers should be considered for efficient light utilization. The assembled configuration is more suitable to further reduce the cost of the system than the tandem one because it does not need wires to connect them. Overall, the STH efficiency of unbiased PEC systems is still lower than that of PV-EC systems; thus, it should be further improved for the real application of large-scale PEC systems.

### 2.3. Particulate PC System

Particulate PC systems do not need membranes and wires, unlike PV-EC and PEC systems. It is also possible to mitigate the main challenges of large-scale PEC systems, such as the large sheet resistance and the limited ionic conductivity. Therefore, it is a cost-feasible pathway for large-scale solar hydrogen production systems. In general, semiconductors with a wide band gap above 3.0 eV, such as TiO_2_ [71] and SrTiO_3_ [72], have been widely used as photocatalysts. They utilize only UV light, thereby showing low STH efficiency. The limited light utilization is enhanced by doping [73,74] and the plasmonic effect by the integration with metal particles [75]. However, the introduction of visible light-responsive semiconductors such as zinc/cadmium sulfide [76,77], carbon nitride [78], and indium gallium nitride [79] is a more fundamental solution to enhance the limited light utilization. The Z-scheme strategy using two semiconductors with different band gaps is also a promising route for improving light utilization [38]. However, it needs an efficient charge mediator in this strategy.

**Figure 8:**
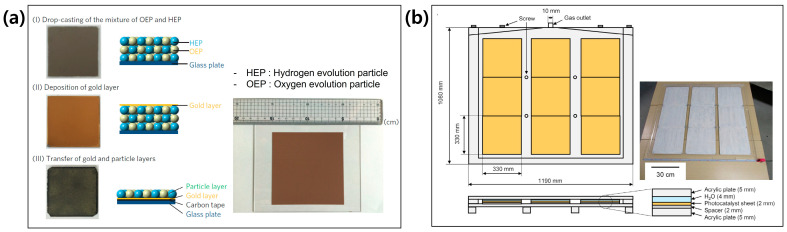
Large-scale PC sheet and panel (**a**) Z-scheme-based PC sheet including La/Rh co-doped SrTiO_3_ HER catalysts and Mo-doped BiVO_4_ OER catalysts with Au charge mediators. Reprinted from [81] with permission from Macmillan Publishers Limited, copyright 2016. (**b**) Al-doped SrTiO_3_ PC panel. Reprinted from [82] with permission from Elsevier Inc., copyright 2018.

Dispersion of photocatalyst particles in the aqueous solution is a traditional method for particulate PC systems [80]. The main limitation of this system is a decrease in light utilization by the suspension of PC powders. Moreover, a huge amount of water is necessary for the large-scale application. PC panels or sheets are a prominent type for overcoming these drawbacks in the traditional particulate PC system. Wang et al. fabricated a Z-scheme-based PC sheet including La/Rh co-doped SrTiO_3_ HER catalysts and Mo-doped BiVO_4_ OER catalysts with Au charge mediators using a particle transfer method, as shown in Figure 8a [81]. They successfully demonstrated a 100 cm^2^ PC sheet with an STH efficiency of 0.1% under continuous operation for 13 h. Goto et al. developed a meter-scale Al-doped SrTiO_3_ PC panel (1 m^2^), as shown in Figure 8b [82]. It was fabricated by a drop-casting method with a SiO_2_ inorganic binder for strong adhesion of photocatalyst particles to the substrate. It was tilted for efficient light absorption and gas release during the operation. In addition, the hydrophilic surface of the panel window facilitated an easy release of generated gases from the PC panel. Its STH efficiency was achieved up to 0.4% under natural sunlight with an intensity of 0.65~0.75 sun. Zhang et al. deposited amorphous MoS_x_-modified CdS particles on Ti mesh substrate by electrodeposition [83]. They successfully upscaled the CdS PC sheet up to 10 × 10 cm^2^. It provided sufficient active sites for HER reaction and anchoring sites for strong adhesion of photocatalyst particles, thereby improving the PC capability in large-scale applications.

**Figure 9:**
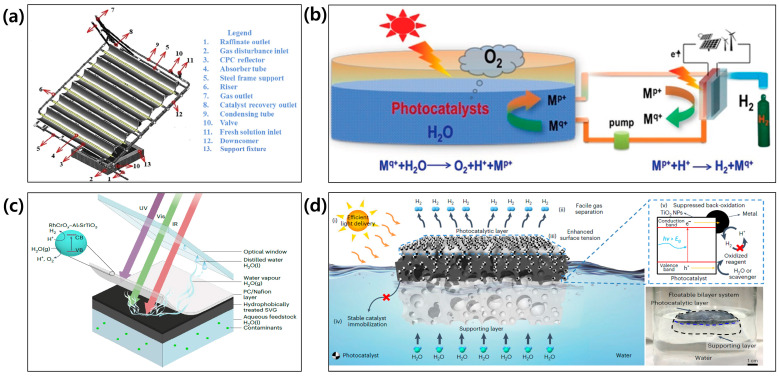
Various concepts and reactors for demonstration of large-scale particulate PC systems. (**a**) Compound parabolic collector (CPC) tubular reactor. Reprinted from [84] with permission from Elsevier Ltd., copyright 2016. (**b**) Hydrogen farm project with the redox shuttle ions. Reprinted from [86] with permission from Wiley-VCH Verlag GmbH & Co. KGaA, Weinheim, copyright 2020. (**c**) Tandem architecture of hybrid PC sheet including solar vapor generator. Reprinted from [87] with permission from The Authors, copyright 2023. (**d**) Floatable photocatalytic platform with porous elastomer-hydrogel composites. Reprinted from [88] with permission from Springer Nature Limited, copyright 2023.

Various concepts and reactors have been suggested for the demonstration of large-scale particulate PC systems. A compound parabolic collector (CPC) tubular reactor is well-known for the large-scale particulate PC system [84,85]. It consists of CPC reflectors for collecting concentrated sunlight and Pyrex tubes for the PC water-splitting reactions (Figure 9a). The continuous water flow is essential to avoid the accumulation of photocatalyst particles in the tubes. Nevertheless, the accumulation or aggregation of particles is inevitable when the size of particles is larger than 10 μm, resulting in the reduction of light utilization. This phenomenon is not observed in the particulate PC system based on sheets and panels. The hydrogen farm project proposed by Zhao et al. was a novel design of a panel-based PC system (Figure 9b) [86]. It did not need a gas separation process because the generated oxygen was directly released into the air due to its unsealed design, unlike CPC or other panel-type reactors. However, the water oxidation process should be improved by suppressing the oxidation of reduced shuttle ions for an efficient system, because its operation mechanism was based on redox shuttle ions. Pornrungroj et al. suggested a hybrid design combining a PC and solar vapor generator (SVG) [87]. It was composed of a SrTiO_3_-based PC sheet as a top layer and a carbon-based SVG component as a bottom layer, as illustrated in Figure 9c. SVG components continuously provided water vapor to the PC sheet by solar heat. The water was efficiently split in a vapor phase, compared to a liquid phase, because of the lower Gibbs free energy and the reduced mass transfer resistance. Lee et al. developed a floatable photocatalytic platform with porous elastomer-hydrogel composites, as illustrated in Figure 9d [88]. The porous elastomer-hydrogel composite not only kept the device afloat but also supplied the water into the PC layer efficiently. Furthermore, it prevented the back oxidation of generated hydrogen by the facile gas separation. They successfully upscaled this device up to 0.0132 m^2^, which produced hydrogen with an evolution rate of 0.504 L/m^2^ per hour.

Table 4 shows the performance of large-scale particulate PC systems. Contrary to the PV-EC and PEC systems, it generally operates in the aqueous solution with sacrificial reagents such as methanol, formic acid, triethanolamine, and lactic acid. It facilitates the fast charge separation and the low charge recombination, thereby improving the STH efficiency. Nevertheless, the STH efficiency is quite lower than PV-EC and PEC systems. Interestingly, some particulate PC systems operate under limited light illumination because they consist of PC materials with a wide band gap. It is also one of the reasons for the low STH efficiency of particulate PC systems. Therefore, the further improvement of STH efficiency is the prime task for the commercialization of particulate PC systems.

### 2.4. Demonstration of Large-Scale Solar Hydrogen Production Systems

**Figure 10:**
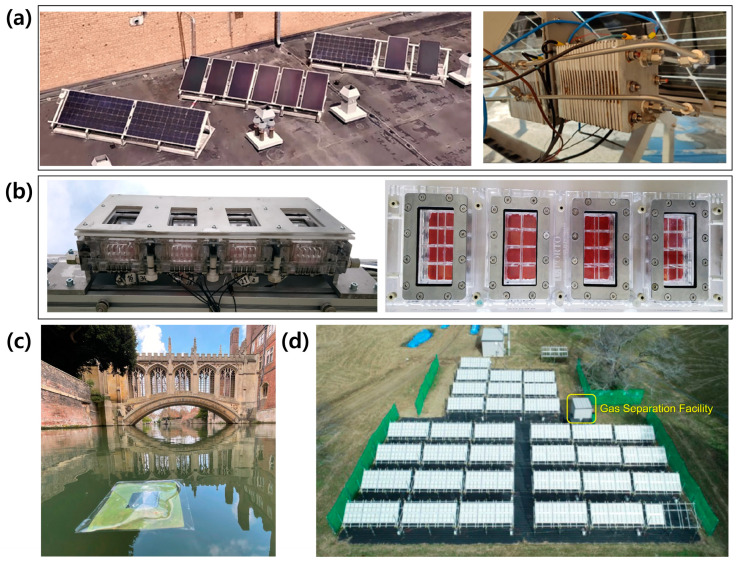
Demonstration of large-scale solar hydrogen production systems under real-world conditions. (**a**) A 10 m^2^ PV-EC system with the combination of CIGS/Si PV modules and PEM electrolyzer in the PECSYS project. Reprinted from [17] with permission from The Authors. Solar RRL published by Wiley-VCH GmbH, copyright 2022. (**b**) A 200 cm^2^ PEC-PV system consisting of multi-Fe_2_O_3_ photoanodes installed in a segmented support reactor and Si heterojunction with intrinsic thin layer PV modules in the PECDEMO project. Reprinted from [67] with permission from Elsevier Ltd., copyright 2020. (**c**) Artificial leaf PEC-PEC system with perovskite photocathode and BiVO_4_ photoanode. Reprinted from [68] with permission from Springer Nature Limited, copyright 2022. (**d**) A 100 m^2^ Al-doped SrTiO_3_ PC panel arrays. Reprinted from [89] with permission from Springer Nature Limited, copyright 2021.

Demonstration of large-scale solar hydrogen production systems has been carried out under outdoor conditions by several research groups and projects. The PECSYS project was aimed at demonstrating a meter-scale PV-EC system. In the initial stage of the project, a 294 cm^2^ PV-EC system with three single heterojunction Si PV modules and NiMo HER/NiFe OER catalysts was demonstrated [37]. It showed a fluctuating STH efficiency (3.4~10%) depending on the temperature and the irradiation intensity during the outdoor measurement. Finally, the PECSYS consortium successfully demonstrated a 10 m^2^ PV-EC system with the combination of CIGS/Si PV modules and PEM electrolyzers (Figure 10a) [17]. It showed an average STH efficiency of 10% and excellent stability with a performance degradation of less than 10% during the operation for 9 months.

Stand-alone systems without external bias, such as PEC-PV and PEC-PEC, have mainly been demonstrated in the PEC system. A 200 cm^2^ PEC-PV system was also successfully demonstrated under naturally concentrated sunlight in the PECDEMO project [67]. It consisted of multi-Fe_2_O_3_ photoanodes installed in a segmented support reactor and Si heterojunction with intrinsic thin-layer PV modules. Sunlight was concentrated by the SoCRatus solar concentrator located in Cologne, Germany. It produced 72 mg of hydrogen during the operation for 13.5 h. The temperature of the system was controlled to avoid the degradation of performance owing to the heat from the concentrated sunlight during the demonstration. An artificial leaf PEC-PEC system suggested by Reisner’s group was successfully tested under real-world conditions on the River Cam at St John’s College in the UK [68]. It was a 100 cm^2^ floating device consisting of a perovskite photocathode and BiVO_4_ photoanode. It produced 100 μmol of hydrogen under weak sunlight illumination (0.2 sun) during the continuous operation for 2 h.

A pilot-scale CPC reactor for large-scale particulate PC systems was demonstrated for a long-term period at Xian in northwest China [92]. It was installed with the ease-west orientation to obtain sufficient solar irradiation above 8 h per day. Daily hydrogen production was periodic in the summer, while it was fluctuant in the spring, due to the seasonal solar irradiation. Consequently, this system using Cd_0_._5_Zn_0_._5_S PC particles produced 36.1 L of hydrogen per day in the summer week, while it produced 20.5 L of hydrogen per day in the spring week on average. A 100 m^2^ Al-doped SrTiO_3_ PC panel was also demonstrated by Domen’s group [89]. An arrayed panel reactor consisting of 33.3 modules with an area of 3 m^2^ was installed at the Kaikioka Research Facility at the University of Tokyo in Japan. It was tested for 3 months in the fall/winter periods. Its STH efficiency was 0.5% in the initial stage of the demonstration, but it was reduced to 0.1% after the operation for 3 months. It was caused by the detachment of photocatalyst particles from the panel due to the repeated freezing and melting of the water. In short, the performance and stability of large-scale solar hydrogen production systems are highly sensitive to harsh weather conditions such as heat and cold waves. Therefore, weathertight designs and concepts are necessary for the durable operation of the efficient solar hydrogen production system.

## 3. Outlook and Perspectives on Real-Life Application

Most PV-EC systems use Si-based PV, which has a dominant market share in the PV market, as a source of water splitting. Perovskite PV has recently emerged as an alternative to Si PV due to its high efficiency and economic feasibility. However, the low efficiency in the large area perovskite PV modules and the degradation in the high temperature/humidity conditions should be overcome for further utilization. In the near future, the state-of-the-art Si/perovskite tandem PV would be the center of attention as a PV source in the PV-EC system because it provides a sufficient photovoltage above 1.9 V without the connection in series for the overall water splitting [93,94].

In the PEC system, protection and passivation strategies are essential for sustainable solar hydrogen production because most semiconductors are unstable in the aqueous solution. Although the atomic layer deposited TiO_2_ is a well-known protection layer for protecting PEC electrodes, the development of alternative deposition methods or the modification of the deposition process is significant because the atomic layer deposition is not suitable for the large area electrode. Research trends on the PEC system move toward the unbiased system, especially the PEC-PEC system because they can produce hydrogen using only sunlight. It provides a less energy-consume process for producing green hydrogen. In this system, the functional membrane is necessary to efficiently operate the system because the HER reaction on the photocathode is active in the acidic solution, while the OER reaction on the photoanode is active in the alkaline solution. Bipolar membrane would be the best option to this end because it operates in both conditions [95].

The particulate PC system is not suitable for large-scale applications because it requires a huge tank and continuous stirring system to disperse PC powders in the aqueous solution. In addition, the accumulation of PC powders on the reactor hinders efficient light utilization. Therefore, research trends on the particulate PC system move to the panel or sheet type in the practical application. However, it is still challenging to apply this system for the mass production of hydrogen due to the lowest STH efficiency among the three solar hydrogen production systems. Furthermore, efficient gas separation techniques are essential to collect pure hydrogen from the generated oxyhydrogen gas without any safety issues. Recently, the integration with water purification, disinfection, and seawater desalination systems has been proposed by several groups [75,85,87]. It would expand the practical availability of particulate PC systems into a multipurpose system in the future.

Overall, attention should be paid to evaluating the STH efficiency of solar hydrogen production systems because the calculation of STH efficiency is different depending on the system type. In general, the STH efficiency is calculated using the produced hydrogen flow in the PV-EC and particulate PC systems, while it is calculated using the operating current density in the PEC systems [34]. It is possible to overestimate the STH efficiency using the latter method when the system has charge losses. Therefore, it would be necessary to establish a reliable evaluation protocol for comparing the technical level of solar hydrogen production systems. In addition, the integration with hydrogen utilization systems such as fuel cells paves the way for developing the compact hydrogen production-utilization merged system toward a hydrogen fuel-based economy.

## 4. Conclusions

Upscaling of the solar hydrogen production system is essential for the mass production of eco-friendly hydrogen. However, there are some challenges in the large-scale solar hydrogen production system to be competitive with the traditional hydrogen production system based on fossil fuels. First, the relatively low STH performance in the large-scale system, especially PEC and particulate PC systems, should be further improved, up to a commercially available level of at least 10%. Second, long-term operation should be viable for sustainable hydrogen production. To this end, suitable protection and passivation strategies on PVs and PEC photoelectrodes are a prerequisite. Third, the economic feasibility of systems should be considered. The replacement of inexpensive materials in the HER/OER catalysts is a promising route for reducing the manufacturing cost of the system. The compact and membrane-less/wireless design is also a useful option for this purpose. Such efforts on the development of efficient and durable large-scale solar hydrogen production will bring us closer to its commercialization in the near future.

## Figures and Tables

**Table 1:** Performances of large-scale PV-EC system: STH efficiency, operational duration, and hydrogen production rate. Values in the brackets are performances from the field test in outdoor conditions. Light condition means the light intensity (1 sun = 1000 W/m^2^, Light illuminations are from the solar simulator using a Xenon lamp with an Air Mass (AM) 1.5 filter, if there are no additional comments. The light intensity is an average value from natural sun during the operation in the outdoor condition).

PV	HER Catalysts	OER Catalysts	Electrolyte	Light Condition	Area	STH Efficiency (%)	Operational Duration	Hydrogen Production	Reference
CIGS + a-Si/c-Si	NiMo	NiFe	DI water	-	10 m^2^	(10)	(6480 h) (10% degraded)	(2.23 g/h·m^2^)	[17]
a-Si:H/μc-Si:H	Ni	Ni	1 M KOH	1 sun	64 cm^2^	3.9	3 h	-	[19]
c-Si	Pt	IrO_2_	1 M KOH	1 sun	5.7 cm^2^	14.2	100 h	-	[20]
a-Si:H/a-Si:H/μc-Si:H	Pt/Ti	IrO_2_/Ti	1 M H_2_SO_4_	1 sun	64 cm^2^	4.8	-	-	[21]
a-Si:H/a-Si:H/μc-Si:H	NiMo/Ni	NiFeO_x_/Ni	1 M KOH	1 sun	64 cm^2^	5.1	10 min	-	[22]
a-Si:H/a-Si:H/μc-Si:H	NiFeMo	NiFeMo	1 M KOH	1 sun	64 cm^2^	4.67	10 min		[23]
Ag-doped CIGS	NiMoV	NiO	1 M KOH	1 sun	100 cm^2^	8.5	100 h	3.7 mL/min	[24]
Ag-doped CIGS	NiFe LDH *	NiFe LDH	1 M KOH	1 sun (Av. 0.8 sun)	82.3 cm^2^	11.3 (9)	- (500 h)	5.74 mL/min (-)	[25]
III-V PV	Pt	IrO_2_	DI water **	(~800 sun)	142.4 cm^2^	(20.3)	(13 days)	(0.8 g/min)	[28]
Perovskite	Graphite	Graphite	KCl	1 sun	12.6 cm^2^	-	-	0.3 mL/min	[29]
c-Si	Ni/SnS_2_/aramid	Ni/SnS_2_/aramid	1 M NaOH	1 sun	7.5 cm^2^	13.5	120 h	-	[32]
c-Si	Pt/Ti	Pt/Ti	0.5 M H_2_SO_4_	1 sun	14.4 cm^2^	5.3	70 min	-	[33]
a-Si/c-Si	Pt	Ir	DI water	(Av. 1 sun)	730 cm^2^	(13.5)	(55 h)	(4.2 g/h·m^2^)	[34]
Perovskite/Si	Pt/C	Ir black	DI water	(Av. 0.39 sun)	342 cm^2^	(6.3)	(72 h)	(0.97 L/h)	[35]
c-Si	Ni	Ni	1 M KOH	0.73 sun, Halogen lamp	154 cm^2^	10.01	20 h	2.23 g/h·m^2^	[36]
c-Si	NiMo	NiFe	1 M KOH	(0.6~0.85 sun)	294 cm^2^	(3.4~10)	-	-	[37]

* LDH: Layered Double Hydroxide, ** DI water: Distilled water.

**Table 2:** Performances of large-scale photoelectrodes for PEC system: photocurrent density biased at HER/OER potentials and operational duration. Values in the brackets are performances from the field test in the outdoor conditions under natural sun. Light condition means the light intensity (1 sun = 1000 W/m^2^, Light illuminations are from the solar simulator using a Xenon lamp with an AM 1.5 filter if there are no additional comments. The light intensity is an average value from natural sun during the operation in the outdoor condition).

Photoelectrodes	Electrolyte	Light Condition	Area (cm^2^)	J_ph_ * (mA/cm^2^)	Operational Duration	Reference
CoPi/W:BiVO_4_ photoanode	2.0 M KPi (pH 7)	1 sun	50	1.5	-	[39]
NiFe/BiVO_4_ photoanode	0.1 M potassium borate (pH 9.35)	1 sun	41.18	2.23	5 h (4% degraded)	[40]
CoPi/BiVO_4_/WO_3_ photoanode	0.1 M phosphate buffer (pH 6.9)	1 sun	25	2.3	80 h	[41]
RuO_x_/TiO_2_/AZO/Cu_2_O photocathode	0.1 M KH_2_PO_4_ + 0.5 M Na_2_SO_4_ (pH 5)	1 sun, LED lamp	50	3.1	60 min	[45]
NiFeOOH/W-BiVO_4_ photoanode	1 M potassium borate (pH 9.3)	1 sun	100	2.10	-	[47]
CoPi/Mo-BiVO_4_/WO_3_ photoanode	0.1 M KPi (pH 7)	1 sun	25	0.74	30 min (50% degraded)	[51]
NiFeO_x_/BiVO_4_ photoanode	0.2 M/Na_2_SO_3_	1 sun	25	4.1	-	[52]
NiFeO_x_/BiVO_4_ photoanode	0.1 M potassium borate (pH 9)	1 sun	100	0.8	-	[53]
Fe_2_O_3_ photoanode	1 M KOH	25 sun, Sulfur plasma lamp	28	0.52	-	[57]
LaTiO_2_N photoanode	0.1 M Na_2_SO_4_ (pH 13.4)	1 sun	40	0.56	110 min (24% degraded)	[61]
CoPi/Ta_3_N_5_ photoanode	0.2 M KH_2_PO_4_ (pH 13)	1 sun	6.25	3	-	[62]
NiOOH/BiVO_4_ photoanode	0.5 M Na_2_SO_4_ (pH 5.5)	1 sun	56.25	0.18	4 h	[63]
CoPi/BiVO_4_ photoanode	0.15 M citric acid (pH 7, with hole scavenger)	1 sun	100	3.55	-	[64]
Pt/TiO_2_/pn^+^Si photocathode	1 M HClO_4_ (pH 0)	(0.85 sun)	(256)	(21.48)	(4 days) (7.9% degraded)	[65]
Hf modified Fe_2_O_3_ photoanode	1 M NaOH (pH 13.6)	1 sun	15.75	0.81	-	[66]

* J_ph_: Photocurrent density biased at 1.23 V versus reverse hydrogen electrode (RHE) in the photoanode or 0 V versus RHE in the photocathode.

**Table 3:** Performances of large-scale unbiased PEC system (PEC-PV and PEC-PEC): STH efficiency, operational duration, and hydrogen production rate. Values in the brackets are performances from the field test in the outdoor conditions under natural sun. Light condition means the light intensity (1 sun = 1000 W/m^2^, Light illuminations are from the solar simulator using a Xenon lamp with an AM 1.5 filter if there are no additional comments. The light intensity is an average value from natural sun during the operation in the outdoor condition).

Configuration	Component 1	Component 2	Electrolyte	Light Condition	Area	STH Efficiency (%)	Operational Duration	Hydrogen Production	Reference
PEC-PV	PEC	PV							
Tandem	CoPi/W:BiVO_4_ photoanode	c-Si	2.0 M KPi	1 sun	50	1.9	7 h	-	[39]
	Dual CoPi/W:BiVO_4_ photoanode					2.1			
Side-by-side	WO_3_ photoanode	DSSC *	0.5 M H_2_SO_4_	1 sun	130.56	-	-	3 mL/min	[44]
Side-by-side	NiFeOOH/W:BiVO_4_ photoanode	c-Si	1 M KBi	1 sun	100	2.2	6 h	-	[47]
Tandem	NiFeO_x_/BiVO_4_ photoanode	c-Si	0.1 M KBi	1 sun	25	1.67	-	-	[53]
Side-by-side				1 sun	25	1.52	-		
Assembled				(Av. 0.8 sun)	225	(1.42)	(40 days)		
Tandem	Fe_2_O_3_ photoanode with NiOOH auxiliary electrodes	c-Si	1 M NaOH	1 sun	200	0.68	8.3 h	-	[59]
Tandem	Ti doped Fe_2_O_3_ photoanode	HIT ** Si	1 M KOH	(Av. 10 sun)	200	(0.12)	(13.5 h)	(5.33 mg/h)	[67]
Tandem	BiVO_4_ photoanode	Perovskite	0.1 M KPi + 0.5 M Na_2_SO_4_	1 sun	11.25	6.56	1000 s	-	[66]
Assembled						6.77			
PEC-PEC	Photoanode	Photocathode							
Tandem	BiVO_4_	Pt/TiO_2_/CdS/Cu_3_BiS_3_	0.2 M NaHPO_4_/NaH_2_PO_4_	1 sun	25	0.47	60 h (10% degraded)	-	[50]
Assembled	TiCo/BiVO_4_	Pt/Perovskite	River water	(Av. 0.2 sun)	100	-	(2 h)	(50 μmol/h)	[68]
Tandem	TiCo/BiVO_4_	Pt/Perovskite	0.1 M KBi + 0.1 M K_2_SO_4_	1 sun	10	0.15	14 h	-	[69]
Side-by-side	NiFe LDH ***/Ni/Perovskite	NiMo/Ni/Perovskite	1 M KOH	(Av. 0.91 sun)	4	(3.4)	(1 h)	-	[70]
Side-by-side	NiFe LDH/Ni/Perovskite	Pt/Perovskite	0.5 M KPi	1 sun	30	9.89	24 h (21% degraded)	146.56 μmol/h	[56]
					100	3.09	7 h (22% degraded)	-	

* DSSC: Dye-Sensistized Solar Cells, ** HIT: Heterojunction with Intrinsic Thin layer, *** LDH: Layered Double Hydroxide.

**Table 4:** Performances of large-scale particulate PC system: STH efficiency, operational duration, and hydrogen production rate. Values in the brackets are performances from the field test in outdoor conditions. Area means a reactor volume in the powder type, while an area of sheet/panel in the sheet/panel type. Light condition means the light intensity (1 sun = 1000 W/m^2^, Light illuminations are from the solar simulator using a Xenon lamp with an AM 1.5 filter if there are no additional comments. The light intensity is an average value from natural sun during the operation in the outdoor condition).

Type	Materials	Electrolyte	Light Condition	Volume /Area	STH Efficiency (%)	Operational Duration	Hydrogen Production	Reference
Powder	Pd/TiO_2_	Water + Methanol	(Av. 0.5 sun)	40 mL	-	-	(32 mmol/h·g)	[71]
Sheet				4.69 cm^2^			(104 mmol/h·g)	
Powder	Au/TiO_2_	0.05 M formic acid	(Av. 0.67 sun)	25 L	-	(5 h)	(6500 μmol/L)	[75]
Powder	Pd/Cd_0_._79_Zn_0_._21_S	0.3 M Na_2_S + 0.2 M Na_2_SO_3_	LED	100 mL	-	3 h	85.67 mL/h	[76]
Powder	Cu/graphdiyne/Cd_0_._5_Zn_0_._5_S	River water	LED	7 L	-	84 h	3.97 μmol/h	[77]
Panel	Pt/g-C_3_N_4_	Water + Triethanolamine	(>0.25 sun)	0.756 m^2^	(0.12)	(30 days)	(0.6 L/day)	[78]
Sheet	Rh/Cr_2_O_3_/Co_3_O_4_/ InGaN/GaN nanowire	DI Water	(160 sun)	16 cm^2^	(6.2)	(140 min)	(15 mmol/h∙cm^2^)	[79]
Powder	ZnS/CdS	0.3 M S^2−^ + 0.2 M SO_3_^2−^	1.2 sun	90 mL	-	-	0.21 mmol/h	[80]
Sheet	La:Rh-SrTiO_3_/Mo-BiVO_4_	Pure water	Xe lamp with cutoff filter (>420 nm)	100 cm^2^	0.1	13 h	173 μmol/h	[81]
Panel	Al:SrTiO_3_	DI water	(0.65~0.75 sun)	1 m^2^	(0.4)	(30 min)	(16.9 mL/min)	[82]
Sheet	MoS_x_/CdS	0.25 M Na_2_S + 0.35 M Na_2_SO_3_	Xe lamp with cutoff filter (>420 nm)	12.25 cm^2^	-	-	17,413 μmol/h·g	[83]
Panel	BiVO_4_	32 mM Fe(NO_3_)_3_	-	1 m^2^	(1.85)	-	-	[86]
Sheet	RhCrO_x_/Al:SrTiO_3_	River water	(Av. 0.6 sun)	25 cm^2^	(0.08)	(2 h)	(7.82 mmol/h·m^2^)	[87]
Panel	Pt/TiO_2_	Water + Methanol	1 sun	0.0132 m^2^	-	14 days	0.504 L/h·m^2^	[88]
	Single atom Cu/TiO_2_		(0.7 sun)	1 m^2^		(12 h)	(79.2 mL/day)	
Panel	Rh/Cr/Co/Al:SrTiO_3_	DI water	(0.2~1 sun)	100 m^2^	(0.5~0.1)	(3 months)	(42 L/h)	[89]
Powder	COF * based TiO_2_	Water + Methanol	(0.01~0.04 sun)	-	(0.24)	(3 days)	(5 μmol/h·g)	[90]
Panel	Pt/CdS on porous microreactor	Water + lactic acid	Xe lamp with cutoff filter (>420 nm)	1 m^2^	-	100 h	602.5 mmol/h·m^2^	[91]

* COF: Covalent Organic Framework.

## Data Availability

The data presented in this study are available upon request from the corresponding author.
